# Associations between hepatitis B virus infection and risk of colorectal Cancer: a population-based prospective study

**DOI:** 10.1186/s12885-021-08846-w

**Published:** 2021-10-18

**Authors:** Tong Liu, Wenqiang Li, Youcheng Zhang, Sarah Tan Siyin, Qi Zhang, Mengmeng Song, Kangping Zhang, Siqing Liu, Hanping Shi

**Affiliations:** 1 Department of Gastrointestinal Surgery/Clinical Nutrition, Capital Medical University Affiliated Beijing Shijitan Hospital, Beijing, 100038 China; 2 Beijing International Science and Technology Cooperation Base for Cancer Metabolism and Nutrition, Beijing, 100038 China; 3 Key Laboratory of Cancer FSMP for State Market Regulation, Beijing, 100038 China; 4 Department of General Surgery, Aerospace Center Hospital, Beijing, China; 5 Department of Hepatobiliary Surgery, The People’s Hospital of Liaoning Province, Shenyang, China; 6 Department of graduate school, Dalian Medical University, Dalian, China; 7 Department of General Surgery, Beijing Children’s Hospital, National Center for Children’s Health, Beijing, China; 8 Department of Hepatological Surgery, Kailuan General Hospital, Tangshan, China

**Keywords:** Hepatitis B virus, Incidence, Colorectal cancer, Competing risk models, Cohort

## Abstract

**Background:**

Previous studies have observed a close association between hepatitis B virus (HBV) infection and hepatocellular carcinoma (HCC) as well as extrahepatic cancers. However, research concerning the effect of HBV infection on the risk of colorectal cancer (CRC) is rare and inconsistent. This study aims to determine the relationship between HBV infection and new-onset CRC.

**Methods:**

We prospectively examined the relationship between HBV infection and new-onset CRC among 93,390 participants from Kailuan Cohort study. Cox proportional hazards regression models, subgroup analyses and competing risk analyses were used to evaluate the association between HBV infection and the risk of new-onset CRC.

**Results:**

During a median follow-up of 11.28 years, 448 incident CRC cases were identified. The adjusted HR (95%confidence interval (CI)) for the association of HBsAg Seropositive with CRC was 1.85(1.15 ~ 2.96) in the Cox regression. Subgroup analyses showed that the HBsAg seropositive group was associated with increased risk of new-onset CRC among male, middle-aged, normal weight, smokers and non-drinker participants, respectively. A positive association of HBV infection with the risk of CRC was observed in the adjusted sub-distribution proportional hazards (SD) models (HR_SD_ = 1.77, 95% CI:1.11–2.84) and cause-specific hazards (CS) models (HR_CS_ = 1.79, 95% CI: 1.13–2.91).

**Conclusions:**

Our results have found a significant association between HBV infection and the risk of incident CRC among Chinese participants.

**Trial registration:**

Kailuan study, ChiCTR–TNRC–11001489. Registered 24 August 2011 - Retrospectively registered, http:// http://www.chictr.org.cn/showprojen.aspx?proj=8050

## Background

The burden of hepatitis B virus (HBV) infection remains a public health threat in many parts of the world. WHO estimates that the global prevalence of HBV infection is 3.5%, with approximately 257 million people currently infected [1]. The majority of HBV-infected people were born before the availability of the hepatitis B vaccine [2]. HBV creates a plasmid-like covalently closed circular DNA (cccDNA) form in the nucleus of infected cells, which leads to chronicity of the HBV infection [3]. HBV infection is estimated to be responsible for 56% of hepatocellular carcinoma (HCC) [4], which is the main histological type of primary liver cancer. More than half of the global incidence and mortality of HCC is in China [1]. Besides HCC, untreated patients with HBV infection are also at an elevated risk for liver fibrosis and liver cirrhosis [5, 6].

A few epidemiological studies have observed a positive relationship between HBV infection and the development of extrahepatic cancers such as non-Hodgkin’s lymphoma, pancreatic cancer, and gallbladder cancer [7–10]. Colorectal cancer (CRC) is the fourth and third most common cancer in men and women respectively [11]. It comprises of almost 10% of global cancer incidence [12]. Well-characterized CRC risk factors include obesity, dietary composition, family history and lack of physical activity [13–15]. Only a few existing studies have demonstrated the positive association of chronic HBV infection and the occurrence of CRC; most studies failed to prove such a link [10, 16]. This discrepancy may be due to different ethnicities or predisposing conditions. Previous studies concerning the association between HBV infection and CRC were conducted in countries with a relatively low prevalence of HBV infection, making it difficult to draw a statistically significant conclusion. Other insufficiencies also included minimal control of confounders, overestimation of CRC incidence among hospitalized participants, lack of assessment of the competing risk events (death) in survival models, and analyses that were rarely stratified by sex or age. The preventive strategies based on prognostic models that estimate the actual individual risk of CRC need to be as accurate as possible, allowing for more appropriate preventive measures and treatments to be implemented quickly and intensively.

Due to the inconsistent results and inadequate evidence, we aimed to clarify the relationship between HBV infection and new-onset CRC by drawing data from Kailuan Study.

## Methods

### Study population

Data were derived from the Kailuan study (Trial identification: ChiCTR–TNRC–11001489; Registration number: 11001489), which is a large, prospective, longitudinal and population-based cohort study. Details regarding the design and methods of the study have been previously described [17, 18]. All corporations’ employees, including retirees, between the ages 18 to 98 years were invited to take part in the baseline physical examinations which took place at Kailuan General Hospital and it’s 10 affiliated hospitals between July 2006 and October 2007. Written consent was obtained from 101,510 participants (65.3%) who agreed to take part. All participants were followed up biennially for collection of information regarding potential risk factors and newly diagnosed CRC cases.

**Fig. 1:**
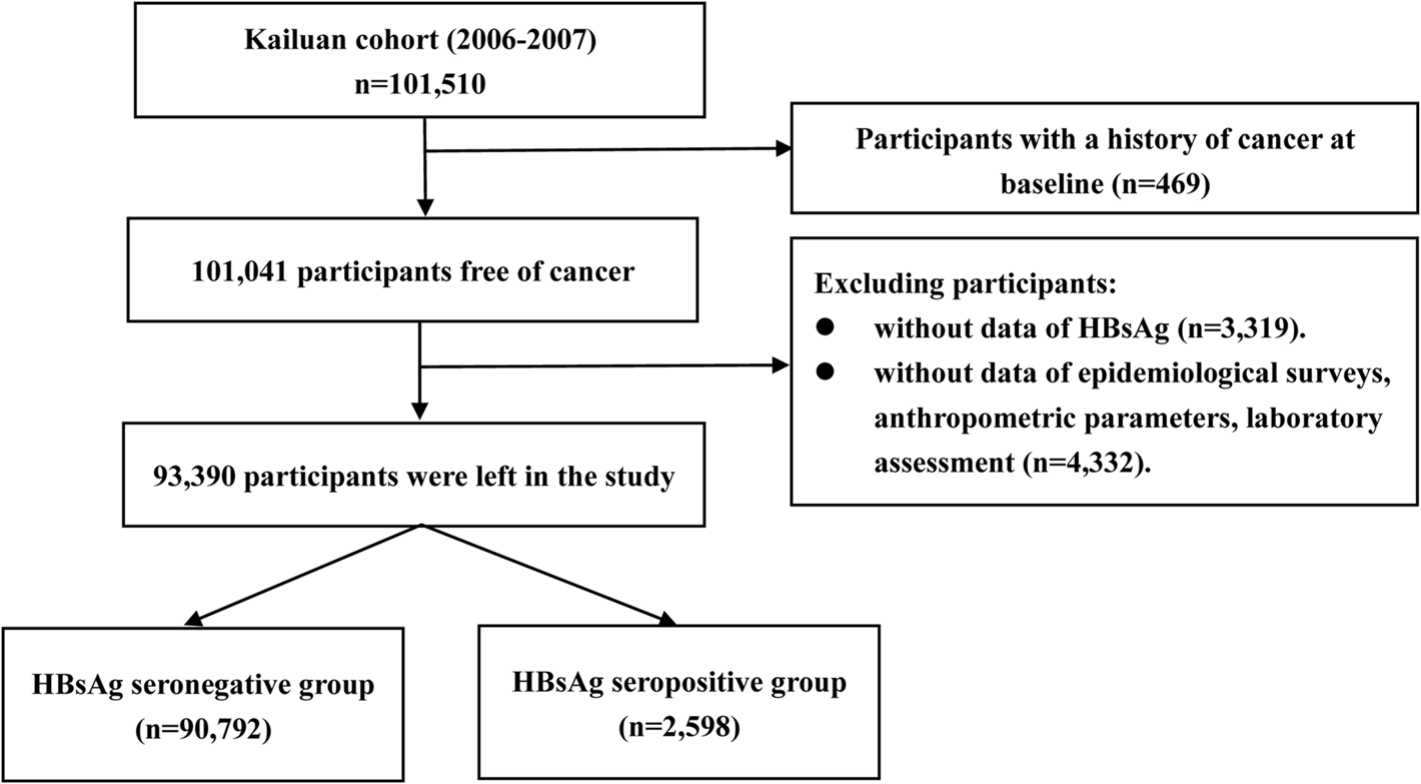
The procedure of participants screening

In the present study, we excluded 469 subjects with a history of cancer at baseline examination, 3319 subjects who had missing data or unclear results for hepatitis B surface antigen (HBsAg), and 4332 subjects without data of other potential variables including age, sex, body mass index (BMI, in kg/m^2^), waist circumference (WC, in cm), systolic blood pressure (SBP, in mmHg), diastolic blood pressure (DBP, in mmHg), total cholesterol (TC, in mmol/L), triglyceride, (TG, in mmol/L), high-sensitivity C-reactive protein (hs-CRP, in mg/L), alanine aminotransferase (ALT, in u/L), total bilirubin (TBil in umol/L), diabetes mellitus, family income, educational background, marital status, salt consumption, current smoker, drinking status, physical activity and family history of cancer. A total of 93,390 subjects were finally enrolled in the study, including 74,644 (79.93%) men and 18,746 (20.07%) women. The details of the participants’ screening were shown in Fig. 1. In this current study, participants who were excluded were younger (49.85 ± 14.13 years versus 51.51 ± 12.44 years, *P* < 0.001), and had lower levels of BMI (24.71 ± 3.45 Kg/m^2^ versus 25.07 ± 3.49 Kg/m^2^, *P* < 0.001), and exhibited lower prevalence of HBV infection (179(2.20%) versus 2598(2.78%), *P* = 0.0105). This study was in compliance with the Declaration of Helsinki and was approved by the Ethics Review Committee of Kailuan General Hospital and Beijing Shijitan Hospital. Written informed consents were obtained from all participants.

### Epidemiological survey and anthropometric parameters

Details of the collection of epidemiological surveys and anthropometric parameters were in accordance with previously published articles [19, 20]. Information on age, sex, lifestyle behaviors, educational background, socioeconomic status, and medical history was recorded via a standard questionnaire which was carried out by the medical staff and trained nurse. BMI was calculated as body weight (kilograms) divided by the square of body height (meters) and categorized into three groups [21]: normal (BMI<24 Kg/m^2^), overweight (24 ≤ BMI<28 Kg/m^2^) and obesity (BMI ≥ 28 Kg/m^2^). Current alcohol consumer was defined as having drunk ≥100 ml/day of alcohol lasting for more than 6 months. Smoking was defined as having 1 cigarette/day at least for more than 6 months. Physical exercise was evaluated from responses regarding the frequency of physical activity (≥3 times/week, ≥30 min/time). Dietary salt intake was self-reported and classified into three categories: low (< 6 g/day), medium (6-9 g/day) or high (≥10 g/day), and high salt diet was defined as consuming salt≥10 g per day on average. Hypertension was defined as: previously diagnosed, and/or a SBP ≥140 mmHg, and/or a DPB ≥90 mmHg, and/or the use of antihypertensive medication [22].

### HBV infection and laboratory assessment

Blood samples were collected using vacuum tubes containing EDTA after an overnight fast (≥ 8 h) from each participant at the baseline. The blood was further centrifuged for 10 min at 3000 rotations per minute at 25 °C. Plasma was separated and stored at − 80 °C until laboratory determinations were performed. An auto-analyzer (Hitachi 747; Hitachi, Tokyo, Japan) was used to analyze all the plasma samples at the Kailuan General Hospital central laboratory. HBsAg was detected quantitatively by the enzyme-linked immunosorbent assay (SHANGHAI KEHUA BIO-ENGINEERING, KHB, Shanghai, China) with standard operating procedure. TC and TG were both measured using enzymatic colorimetric method (Mind Bioengineering Co. Ltd., Shanghai, China). Hs-CRP was measured using a high-sensitivity nephelometry assay (Cias Latex CRP-H, Kanto Chemical Co. Inc). ALT was measured using an enzymatic rate method (Mind Bioengineering Co. Ltd., Shanghai, China). Serum TBil was measured using a chemical oxidation method (MedicalSystem Biotechnology, China). Diabetes mellitus was defined as follows [23]: a fasting blood glucose level ≥ 7.0 mmol/L, taking oral hypoglycemic agents or insulin, or a validated physician diagnosis. According to the Guidelines for the Prevention and Treatment of Adult Dyslipidemia in China [24], hypercholesterolemia and hypertriglyceridemia were defined as TC ≥ 6.2 mmol/L and TG ≥ 2.3 mmol/L, respectively. Hyperbilirubinemia was defined as serum TBil> 21 umol/L.

### Outcome assessment

The follow-up of each participant began at the end of baseline examination and terminated at the occurrence of any event as follows came first: CRC, death, or end of the follow-up (December 31, 2019). Incident CRC and CRC-unrelated death cases were obtained through routine biennial health examinations. Further CRC and CRC-unrelated death cases were assessed annually by checking discharge summaries from Kailuan General Hospital and its 10 affiliated hospitals where participants received treatments. Furthermore, medical records linked with the Tangshan medical insurance system and death certificates from the Kailuan social security system were checked to further confirm the outcomes. CRC cases were coded according to the International Classification of Diseases, Tenth Revision (ICD–10), and CRC was coded as C18-C20.

### Statistical analysis

Person-years of follow-up were calculated from the date of recruitment to the date of incidence, death, or termination of follow-up. Mean (standard deviation, SD) and *t*-test were used to describe and compare continuous variables. The nonparametric Kruskal-Wallis Test and median (interquartile range) were used to describe and test the differences of skewed distribution variables including serum hs-CRP, ALT, TG and TBil between groups. Proportions and Chi-square tests were used to describe and compare categorical variables. Hazard ratios (HRs) and 95% confidence intervals (CIs) were estimated by multivariable Cox proportional hazards regression models to evaluate the association between HBV infection and the risk of new-onset CRC. Model 1 was a univariate analysis. Model 2 was adjusted for age (every 10 years) and sex. Model 3 was further adjusted for BMI (normal, overweight, obesity), hypercholesterolemia, hypertriglyceridemia, hs-CRP (< 1 mg/L, 1–3 mg/L, > 3 mg/L), hyperbilirubinemia, elevated alanine aminotransferase, diabetes, family income, educational background, marital status, salt consumption, current smoker, drinking status, physical activity and family history of cancer based on model 2.

Subgroup analyses were performed by sex (women vs. men), age (young group vs. middle-aged group vs. elder group), BMI (normal weight vs. overweight vs. obesity), smoking status (non-smoker vs. smoker) and drinking status (non-drinker vs. drinker). The interactions between HBV infection status and these variables were further tested by multiplicative models.

During follow-up, CRC unrelated death may occur prior to the occurrence of CRC cases. Due to the existence of competing risk events (CRC unrelated death), the observation of new-onset CRC and further interventions can be hindered. Conventional methods for survival analysis such as standard Cox proportional hazards regression may neglect the competing events and overestimate the absolute risk of the disease [25, 26]. Thus, competing risk analysis should be applied to epidemiologic research. In the current study, cause-specific hazards models (CS models) and sub-distribution proportional hazards models (SD models) were used to calculate HR_CS_ and HR_SD_ of CRC with the existence of competing risk by fitting into the standard Cox proportional hazards regressions.

In the sensitivity analysis, we further excluded 39 participants who had CRC within the first year after entry to the cohort to investigate the possibility of reverse causation.

Statistical computations were performed using a commercially available software program (SAS software, version 9.4). Reported *P*-values are two-sided, and the significance level was set at *p* < 0.05.

## Results

### Characteristics of the study population

The mean ± SD age of the 93,390 participants was 51.52 ± 12.43 years with 74,644 (79.93%) men and 18,746 (20.07%) women. The overall prevalence of HBsAg seropositive was 2.8%. Individuals who were HBsAg seropositive were younger, had lower SBP levels, TC, hs-CRP and TG concentrations. HBsAg seropositive group was also associated with elevated ALT and TBil concentrations. Lower prevalence of hypertension, physical exercise, income, high-school graduation, and higher consumption of tobacco products were also observed in the HBsAg seropositive group. There was no difference in the prevalence of diabetes mellitus, current drinking, family history of cancer, marital status, high salt diets and the levels of BMI, WC and DBP between groups. The baseline characteristics for participants stratified by HBV infection status are shown in Table 1.

**Table 1:** Baseline characteristics of the participants

	HBsAg Seronegative ***n*** = 90,792	HBsAg Seropositive ***n*** = 2598	t/X^**2**^	***P-*** value
**Age (year)**	**51.58 ± 12.46**	**49.23 ± 11.47**	**90.59**	**< 0.0001**
**Male**	**72,418(79.76)**	**2226(85.68)**	**55.15**	**< 0.0001**
**BMI (Kg/m**^**2**^**)**	**25.07 ± 3.49**	**25.06 ± 3.50**	**0.02**	**0.8998**
**WC (cm)**	**86.98 ± 9.96**	**86.71 ± 10.06**	**1.71**	**0.1893**
**SBP (mmHg)**	**131.18 ± 21.10**	**129.47 ± 19.85**	**16.52**	**< 0.0001**
**DBP (mmHg)**	**83.63 ± 11.79**	**83.38 ± 11.63**	**1.12**	**0.2899**
**TC (mmol/L)**	**4.96 ± 1.15**	**4.66 ± 1.18**	**176.61**	**< 0.0001**
**hs-CRP (mg/L)**	**0.80(0.30 ~ 2.09)**	**0.73(0.30 ~ 1.75)**	**121.01**	**< 0.0001**
**TG (mmol/L)**	**1.28(0.90 ~ 1.94)**	**1.15(0.80 ~ 1.61)**	**115.10**	**< 0.0001**
**ALT (u/L)**	**18.11(13.21 ~ 24.80)**	**23.03(16.90 ~ 33.17)**	**595.31**	**< 0.0001**
**TBil (umol/L)**	**12.20(9.80 ~ 15.20)**	**12.70(10.40 ~ 16.40)**	**64.12**	**< 0.0001**
**BMI (%)**			**0.1277**	**0.9381**
**BMI < 24 Kg/m**^**2**^	**35,702(39.32)**	**1027(39.53)**		
**24 ≤ BMI<28 Kg/m**^**2**^	**38,071(41.93)**	**1091(41.99)**		
**BMI ≥ 28 Kg/m**^**2**^	**17,019(18.75)**	**480(18.48)**		
**Hs-CRP (%)**			**15.29**	**0.0005**
**< 1 mg/L**	**50,787(55.94)**	**1532(58.97)**		
**1–3 mg/L**	**23,348(25.72)**	**663(25.52)**		
**> 3 mg/L**	**16,657(18.35)**	**403(15.51)**		
**Hypercholesterolemia (%)**	**10,302(11.35)**	**170(6.54)**	**58.53**	**< 0.0001**
**Hypertriglyceridemia (%)**	**16,651(18.34)**	**320(12.32)**	**61.61**	**< 0.0001**
**Hyperbilirubinemia (%)**	**6036(6.65)**	**242(9.31)**	**28.64**	**< 0.0001**
**Elevated alanine aminotransferase (%)**	**5536(6.10)**	**460(17.71)**	**566.49**	**< 0.0001**
**Hypertension (%)**	**39,913(43.96)**	**1057(40.69)**	**11.01**	**0.0009**
**Diabetes mellitus (%)**	**8338(9.18)**	**218(8.39)**	**1.91**	**0.1674**
**Physical exercise (%)**	**14,358(15.81)**	**353(13.59)**	**9.44**	**0.0021**
**Current smoker (%)**	**28,035(30.88)**	**907(34.91)**	**19.21**	**< 0.0001**
**Current drinker (%)**	**16,332(17.99)**	**443(17.05)**	**1.50**	**0.2200**
**Family history of cancer (%)**	**3320(3.66)**	**102(3.92)**	**1.01**	**0.3135**
**Marital status (married, (%))**	**85,668(94.36)**	**2444(94.07)**	**5.15**	**0.2721**
**High salt diets ((≧ 10 g/day, (%))**	**9796(10.79)**	**299(11.51)**	**1.36**	**0.5077**
**High-school graduation or above (%)**	**17,883(19.70)**	**505(19.44)**	**9.95**	**0.0413**
**Reported income of each family member (≧800¥, (%))**	**12,944(14.26)**	**341(13.13)**	**11.57**	**0.0090**

*BMI* Body mass index, *WC* waist circumference, *SBP* Systolic blood pressure, *DBP* Diastolic blood pressure, *TC* Total cholesterol, *hs-CRP* High-sensitivity C-reactive protein, *TG* Triglyceride, *ALT* Alanine aminotransferase, *TBil* Total bilirubin

### Association of HBV infection with the risk of CRC

During a median follow-up of 11.28 years, 448 incident CRC were identified over a total of 1,071,123 person-years among 93,390 participants. In the univariate analysis, compared with HBsAg Seronegative participants, no significant relationship between HBV infection and risk of CRC was observed (HR = 1.52, 95% CI: 0.95–2.44). After adjustments were made for the potential confounders, participants with HBsAg seropositive had a significantly increased risk of developing incident CRC (HR = 1.85, 95% CI: 1.15–2.96), (Table 2). Table 3 demonstrated the effects of HBV infection on the risk of CRC after stratifying the participants by sex, age, BMI, smoking status and drinking status. Subgroup analyses showed that the HBsAg Seropositive group was associated with a 75% (HR = 1.75, 95% CI: 1.04–2.94), 95% (HR = 1.95, 95% CI: 1.14–3.34), 172% (HR = 2.72, 95% CI: 1.43–5.16), 220% (HR = 3.20, 95% CI: 1.73–5.95) and 126% (HR = 2.26, 95% CI: 1.38–3.68) increased risk of new-onset CRC among male, middle-aged, normal weight, smoker and non-drinker participants, respectively. However, those associations were not observed in participants who were female (HR = 2.31, 95% CI: 0.71–7.50), younger (HR = 2.74, 95% CI: 0.64–11.85), elderly (HR = 0.87, 95% CI: 0.21–3.51), overweight (HR = 1.42, 95% CI: 0.63–3.22), obese (HR = 0.94, 95% CI: 0.23–3.82), non-smokers (HR = 1.05, 95% CI: 0.50–2.22) and alcohol consumers (HR = 0.39, 95% CI: 0.06–2.81). Present results suggest that the interaction of smoking status and HBV infection on the risk of CRC is significant (*P* for interaction = 0.0247). However, no evidence of interaction effect of HBV infection with sex, age, BMI and drinking status was observed.

**Table 2:** The association of HBV infection with the risk of CRC among different regressions

	HBsAg Seronegative		HBsAg Seropositive		Adjusted Hazard Ratios (95% CI)
	Cases	Person-years	Cases	Person-years	
**Multivariate COX Regression**					
**Model 1**	**430**	**1,042,506**	**18**	**28,617**	**1.52(0.95 ~ 2.44)**
**Model 2**	**430**	**1,042,506**	**18**	**28,617**	**1.79(1.12 ~ 2.87)**
**Model 3**	**430**	**1,042,506**	**18**	**28,617**	**1.85(1.15 ~ 2.96)**
**SD Model**					
**Model 1**	**430**	**1,042,506**	**18**	**28,617**	**1.47(0.92 ~ 2.36)**
**Model 2**	**430**	**1,042,506**	**18**	**28,617**	**1.71(1.07 ~ 2.74)**
**Model 3**	**430**	**1,042,506**	**18**	**28,617**	**1.77(1.11 ~ 2.84)**
**CS Model**					
**Model 1**	**430**	**1,042,506**	**18**	**28,617**	**1.50(0.94 ~ 2.40)**
**Model 2**	**430**	**1,042,506**	**18**	**28,617**	**1.74(1.11 ~ 2.78)**
**Model 3**	**430**	**1,042,506**	**18**	**28,617**	**1.79(1.13 ~ 2.91)**

Note:

Model 1: Univariate analysis

Model 2: Adjusted for age (every 10 years), sex based on model 1

Model 3: Further adjusted for BMI (normal, overweight, obesity), hypercholesterolemia, hypertriglyceridemia, hs-CRP (< 1 mg/L, 1–3 mg/L, > 3 mg/L), hyperbilirubinemia, elevated alanine aminotransferase, diabetes, family income, educational background, marital status, salt consumption, current smoker, drinking status, physical activity and family history of cancer based on model 2

*CS model* Cause-specific hazard model, *SD model* Sub-distribution hazard function model

**Table 3:** Stratified analysis of the association of HBV infection with the risk of CRC

	HBsAg Seronegative		HBsAg Seropositive		Adjusted Hazard Ratios (95% CI)	***P*** for interaction
	Cases	Person-years	Cases	Person-years		
**Sex** ^**a**^						**0.6045**
**Women**	**61**	**216,063**	**3**	**4264**	**2.31(0.71 ~ 7.50)**	
**Men**	**369**	**826,442**	**15**	**24,352**	**1.75(1.04 ~ 2.94)**	
**Age (years)** ^**b**^						**0.2749**
**Age ≤ 45**	**20**	**317,261**	**2**	**10,746**	**2.74(0.64 ~ 11.85)**	
**45 < Age ≤ 65**	**280**	**591,855**	**14**	**15,537**	**1.95(1.14 ~ 3.34)**	
**Age > 65**	**130**	**133,340**	**2**	**2334**	**0.87(0.21 ~ 3.51)**	
**BMI (Kg/m**^**2**^**)** ^**c**^						**0.1009**
**BMI < 24**	**154**	**408,823**	**10**	**11,149**	**2.72(1.43–5.16)**	
**24 ≤ BMI<28**	**186**	**438,055**	**6**	**12,174**	**1.42(0.63 ~ 3.22)**	
**BMI ≥ 28**	**90**	**195,626**	**2**	**5292**	**0.94(0.23 ~ 3.82)**	
**Current smoker** ^**d**^						**0.0247**
**No**	**298**	**719,868**	**7**	**18,797**	**1.05(0.50 ~ 2.22)**	
**Yes**	**132**	**322,638**	**11**	**9820**	**3.20(1.73 ~ 5.95)**	
**Current drinker** ^**e**^						
**No**	**321**	**854,353**	**17**	**23,686**	**2.26(1.38 ~ 3.68)**	**0.0892**
**Yes**	**109**	**188,153**	**1**	**4930**	**0.39(0.06 ~ 2.81)**	

Note:

All models were adjusted for hypercholesterolemia, hypertriglyceridemia, hs-CRP (< 1 mg/L, 1–3 mg/L, > 3 mg/L), hyperbilirubinemia, elevated alanine aminotransferase, diabetes, family income, educational background, marital status, salt consumption, physical activity and family history of cancer

^a^Age (every 10 years), BMI (normal, overweight, obesity), current smoker and drinking status were further adjusted when participants were stratified by sex

^b^Sex, BMI (normal, overweight, obesity), current smoker and drinking status were further adjusted when participants were stratified by age. Age was also adjusted within each age stratum to prevent residual confounding

^c^Age (every 10 years), sex, current smoker and drinking status were further adjusted when participants were stratified by BMI

^d^Age (every 10 years), BMI (normal, overweight, obesity), sex and drinking status were further adjusted when participants were stratified by smoking status

^e^Age (every 10 years), BMI (normal, overweight, obesity), sex and smoking status were further adjusted when participants were stratified by drinking status

### Association of HBV infection with the risk of CRC in the competing risk analysis

9882 CRC unrelated-death cases were identified during almost 11 years follow-up among 93,390 participants. After adjusting for the potential confounders, including age (every 10 years), sex, BMI (normal, overweight, obesity), hypercholesterolemia, hypertriglyceridemia, hs-CRP (< 1 mg/L, 1–3 mg/L, > 3 mg/L), hyperbilirubinemia, elevated alanine aminotransferase, diabetes, family income, educational background, marital status, salt consumption, current smoker, drinking status, physical activity and family history of cancer, a statistically significant association of HBV infection with the risk of CRC was observed in the adjusted SD models (HR_SD_ = 1.77, 95% CI:1.11–2.84). Similar results were also found in the adjusted CS models (HR_CS_ = 1.79, 95% CI: 1.13–2.91) (Table 2).

### Sensitivity analysis

After excluding 39 CRC cases that occurred during the first year of follow-up, participants with HBV infection were associated with a 68% (HR = 1.68, 95% CI:1.05–2.69) increase in the risk of CRC in the univariate analysis. The association remained statistically significant when the analysis was further adjusting for the potential confounders (HR = 2.05, 95% CI:1.28–3.30; Table 4).

**Table 4:** The association of HBV infection with the risk of CRC (exclude participants who occurred HCC within 1 year)

	HBsAg Seronegative		HBsAg Seropositive		Adjusted Hazard Ratios (95% CI)
	Cases	Person-years	Cases	Person-years	
**Model 1**	**391**	**1,042,341**	**18**	**28,600**	**1.68(1.05 ~ 2.69)**
**Model 2**	**391**	**1,042,341**	**18**	**28,600**	**1.98(1.23 ~ 3.17)**
**Model 3**	**391**	**1,042,341**	**18**	**28,600**	**2.05(1.28 ~ 3.30)**

Note:

Model 1: Univariate analysis

Model 2: Adjusted for age (every 10 years), sex based on model 1

Model 3: Further adjusted for BMI (normal, overweight, obesity), hypercholesterolemia, hypertriglyceridemia, hs-CRP (< 1 mg/L, 1–3 mg/L, > 3 mg/L), hyperbilirubinemia, elevated alanine aminotransferase, diabetes, family income, educational background, marital status, salt consumption, current smoker, drinking status, physical activity and family history of cancer based on model 2

## Discussion

In this large-scale prospective cohort study, we observed a significant association between HBV infection and the risk of incident CRC among Chinese participants. The results were robust and stable even in the competing risk analyses which took CRC unrelated-death as the competing event. Subgroup analyses have shown that the association might be modified by categories of sex, age, BMI, smoking status and drinking status. Sensitivity analyses further confirmed the consistency of our findings. To our knowledge, this is the first prospective cohort study to investigate the relationship between HBV infection and the risk of new-onset CRC among Chinese participants by using competing risk analyses.

The association between HBV infection and the risk of CRC has not been fully documented previously, despite this, our findings are in line with several population-based studies. Results from a population-based study in Taiwan showed that participants with HBV infection could expect a 36% increase in the risk of CRC compared with HBsAg seronegative participants in an analysis adjusted for sex, age, region, occupation, level of urbanization, income and the presence of comorbidities [27]. By analyzing the data of 496,732 participants in the China Kadoorie Biobank (CKB) prospective cohort [28], Ci Song et.al found that HBV infection was positively associated with an elevated risk of CRC (HR = 1.36, 95% CI:1.09–1.70). However, a retrospective chart review failed to find the association between chronic HBV infection and colorectal adenoma [29]. This discrepancy may be due to a retrospective design, and a small sample size.

The association of HBV infection with the risk of CRC can be modified across categories of sex, age, BMI, smoking status and drinking status. Significant associations were not observed in participants who were females, younger, elderly, overweight, obese, non-smokers or alcohol consumers. Several explanations may help to clarify these discrepancies. First, despite the fact that abnormally elevated estrogen concentrations are associated with the increased risk of endometrial carcinoma and breast cancer [30], a meta-analysis observed a consistent protective effect of estrogens against colorectal cancer risk in both case-control studies and cohort studies [31]. The protection of estrogen in the development of CRC may explain the discrepancy between sex groups. Second, CRC is rare among neonates and young people and the cause-effect relationship may yield null results during short-term exposure to HBV infection among these participants. In elderly participants (mean age: 71.53 ± 5.18), long-term exposure to chronic HBV infection could lead to cirrhosis and hepatocellular carcinoma. In this current study, 14 (0.11%) cirrhosis cases were identified among elder participants versus 77 (0.09%) cases in the relatively younger participants (age ≤ 65 years). These long-term complications as well as cardiovascular events and stroke are life-threatening, and thus might hinder the occurrence of CRC. Third, although World Cancer Research Fund (WCRF) and the International Agency for Research on Cancer (IARC) have proved that overweight and obesity increase the incidence of several cancers [32], unexpected inverse relationship between obesity and cancer mortality, the obesity paradox, has also been seen in other cancer types [33]. Previous studies have demonstrated that obesity CRC patients are related to a better prognosis in the metastatic setting [34]. Forth, non-smoker participants have also been associated with healthier life behaviors and lifestyles, factors which might have reversed the impact of the incidence of CRC. Alcohol consumption has been proved to be closely related to colorectal cancer [35]. However, we have no explanation for the negative association between HBV infection and CRC among drinking participants. Further study should be conducted to explore whether alcohol can eliminate the effect of HBV infection on the risk of CRC.

The association of HBsAg Seropositive with the occurrence of CRC is further confirmed by taking CRC unrelated-death as a competing event into the analyses. In the presence of CRC unrelated-death, competing risk will occur when participants experience a different outcome that competes with CRC cases. Epidemiologic research is fraught with competing risks. Conventional approaches for survival analysis which neglect the nature of competing events, such as the Kaplan-Meier method and standard Cox proportional hazard regression, may be inadequate. Alternative methods explicitly intended for evaluating competing risks data should be used. In the presence of competing risks, understanding the type of research that requires answering is crucial when selecting a method for survival analysis. Epidemiological studies generally address two main types of research questions. Risk factors or determinants and a given outcome are studied for aetiological research. The probability of a specific outcome at a specific time for an individual patient is studied for prognostic research. CS and SD models have been proven to be distinct by prior studies; research on disease etiology would be more suited for CS models, while prognostic research predicting an individual’s risk for an outcome or resource allocation would be more suited for SD models [36].

The mechanisms of HBV-induced hepatocellular carcinoma have been well elucidated [37]. However, no existing study has investigated the pathophysiological mechanism of the association between HBV infection and the risk of incident CRC. Due to the persistence of chronic HBV infection, the adaptive immunity changes from immune tolerance to progressive immune activation, inactivation, reactivation and exhaustion, which may pave the way to the development of HBV-associated CRC [38]. In addition, previous studies have detected the existence of HBV in several types of non-liver tissues including pancreas, kidney, skin and gastric mucosa [39, 40]. The integration of HBV DNA into its host genome takes place during the early stages of clonal tumor expansion, which causes genomic instability as well as direct insertional mutagenesis of various cancer-related genes. Research has shown that cancer patients have higher HBX and anti-HBc protein expression when compared to non-cancer patients [37]. Conscripted on cellular chromatin, HBX controls chromatin dynamics at precise gene loci. HBV-related tumors are associated with higher levels of chromosomal abnormalities, p53 inactivation due to mutations, and fetal hepatic progenitor cell gene overexpression than tumors connected to other risk factors [41]. Besides driving liver chronic necro-inflammation and stimulating the host immune response, there is a possibility that HBV is concealed in non-liver cells, and could initiate common and etiology-specific oncogenic pathways [42].

In this large-scale community-based study, one of the most significant strengths is the prospective design, which is less prone to recall bias than retrospective studies and more suited to examine the temporal association between potential risk factors and disease. The large sample size, long follow-up period along with a good number of incident CRC cases increase the accuracy and creditability of the results. In addition, the strengths of our study include an almost 100% follow-up rate via medical records, death certificates and health insurance among target participants, and the broad assessment of potential confounders which has been well addressed in this study. Lastly, although Cox regression was applied to estimate the relative risks of CRC as previous studies [27, 28], the implementation of competing risk regression makes our results more robust in the presence of competing risk.

Several limitations should also be noted when interpreting the results. First, the Kailuan study does not contain detailed information about the consumption of cereal, vegetables and high-fiber food, which hinders us from assessing confounding factors more precisely. Second, there is an imbalance in sex distribution caused by the industrial nature of the Kailuan Community. Nonetheless, since we conducted independent statistical research on both sexes, the impact of imbalance in sex distribution on the results would be minimal. Third, the industrial nature of Kailuan community homes mainly labor workers. Thus, extrapolated results might not be an accurate description of the wider Chinese population. Fourth, although we found an increased risk of CRC in chronic carriers of HBV, future studies should be conducted to detect the existence of HBV in colon tumors by using molecular biology, immunohistochemical methods to better elucidate the pathophysiological explanation of our findings.

## Conclusions

Our results have provided evidence that chronic HBV infection plays an important role in the occurrence of CRC. The main clinical implications would be an increased awareness of early screening for CRC in individuals infected with HBV. We also encourage using competing risk models as a standard tool for developing predictive models in epidemiology studies.

## Data Availability

The data sets generated and/or analyzed in this study are not publicly available. However, they are available from the corresponding author upon reasonable request.

## Abbreviations

HBV: Hepatitis B virus
HCC: Hepatocellular carcinoma
CRC: Colorectal cancer
cccDNA: Covalently closed circular DNA
HBsAg: Hepatitis B surface antigen
BMI: Body mass index
WC: Waist circumference
SBP: Systolic blood pressure
DBP: Diastolic blood pressure
TC: Total cholesterol
TG: Triglyceride
hs-CRP: High-sensitivity C-reactive protein
ALT: Alanine aminotransferase
TBil: Total bilirubin
HRs: Hazard ratios
CIs: Confidence intervals
CS model: Cause-specific hazards model
SD model: Sub-distribution proportional hazards model
